# Triterpene Acids from Frankincense and Semi-Synthetic Derivatives That Inhibit 5-Lipoxygenase and Cathepsin G

**DOI:** 10.3390/molecules23020506

**Published:** 2018-02-24

**Authors:** Andreas Koeberle, Arne Henkel, Moritz Verhoff, Lars Tausch, Stefanie König, Dagmar Fischer, Nicole Kather, Stefanie Seitz, Michael Paul, Johann Jauch, Oliver Werz

**Affiliations:** 1 Department of Pharmaceutical/Medicinal Chemistry, Institute of Pharmacy, Friedrich-Schiller-University, Philosophenweg 14, D-07743 Jena, Germany; andreas.koeberle@uni-jena.de (A.K.); stefanie.koenig@uni-jena.de (S.K.); 2 Department of Pharmaceutical Analytics, Pharmaceutical Institute, Eberhard Karls University, Auf der Morgenstelle 8, D-72076 Tuebingen, Germany; arne.henkel@gmail.com (A.H.); mzverhoff@googlemail.com (M.V.); 3 Institute of Pharmaceutical Chemistry, University of Frankfurt, Max-von-Laue-Str. 9, D-60438 Frankfurt, Germany; l.tausch@yahoo.de; 4 Department of Pharmaceutical Technology, Institute of Pharmacy, Friedrich-Schiller-University, Otto-Schott-Strasse 41, D-07745 Jena, Germany; dagmar.fischer@uni-jena.de; 5 Organic Chemistry II, Saarland University, Campus C4.2, D-66123 Saarbruecken, Germany; dr.nicole.kolz@t-online.de (N.K.); seitz.stefanie@gmx.net (S.S.); m.paulpaul@web.de (M.P.); j.jauch@mx.uni-saarland.de (J.J.)

**Keywords:** triterpene acid, 5-lipoxygenase, cathepsin, frankincense, boswellic acid, microsomal prostaglandin E_2_ synthase

## Abstract

Age-related diseases, such as osteoarthritis, Alzheimer’s disease, diabetes, and cardiovascular disease, are often associated with chronic unresolved inflammation. Neutrophils play central roles in this process by releasing tissue-degenerative proteases, such as cathepsin G, as well as pro-inflammatory leukotrienes produced by the 5-lipoxygenase (5-LO) pathway. Boswellic acids (BAs) are pentacyclic triterpene acids contained in the gum resin of the anti-inflammatory remedy frankincense that target cathepsin G and 5-LO in neutrophils, and might thus represent suitable leads for intervention with age-associated diseases that have a chronic inflammatory component. Here, we investigated whether, in addition to BAs, other triterpene acids from frankincense interfere with 5-LO and cathepsin G. We provide a comprehensive analysis of 17 natural tetra- or pentacyclic triterpene acids for suppression of 5-LO product synthesis in human neutrophils. These triterpene acids were also investigated for their direct interference with 5-LO and cathepsin G in cell-free assays. Furthermore, our studies were expanded to 10 semi-synthetic BA derivatives. Our data reveal that besides BAs, several tetra- and pentacyclic triterpene acids are effective or even superior inhibitors of 5-LO product formation in human neutrophils, and in parallel, inhibit cathepsin G. Their beneficial target profile may qualify triterpene acids as anti-inflammatory natural products and pharmacological leads for intervention with diseases related to aging.

## 1. Introduction

Persistent inflammation is a central component of numerous widespread and devastating chronic diseases, including osteoarthritis, atherosclerosis, cancer, type 2 diabetes, and Alzheimer’s disease [1], that dominate in older people [2]. The pro-inflammatory eicosanoids prostaglandin (PG) E_2_ and leukotrienes (LT), produced by cyclooxygenase (COX) and 5-lipoxygenase (5-LO) pathways from arachidonic acid (AA), as well as secreted proteases from innate immune cells, significantly contribute to the inflammatory response [3]. Non-steroidal anti-inflammatory drugs (NSAIDs) suppress inflammation, essentially by interference with eicosanoid biosynthesis, and they are the most frequently used therapeutics to treat chronic inflammatory diseases [4]. Moreover, NSAIDs are considered as the most commonly self-prescribed class of anti-inflammatory and anti-nociceptive drugs in the elderly population [5]. However, long-term use of NSAIDs is associated with frequent and severe side effects, such as gastric and renal toxicities [4], and they can reduce the efficacy of diuretics and ACE inhibitors, drugs that are commonly used by elderly patients [5].

The gum resin from various *Boswellia* species, termed frankincense, is a traditional Ayurvedic medicine that has experienced increasing popularity also in Western countries during the past decades [6]. Frankincense is a natural and rich source for a variety of triterpene acids, including boswellic acids (BAs), tirucallic acids, roburic acids, and lupeolic acids [7,8,9]. In folk medicine, lipophilic frankincense extracts are used as alternative to anti-inflammatory steroidal drugs (i.e., glucocorticoids) or NSAIDs for treatment of inflammatory diseases, such as rheumatoid arthritis, osteoarthritis, asthma, atopic dermatitis, and inflammatory bowel diseases [6]. The 3-*O*-acteyl-11-keto-β-BA (AKBA **1**), 11-keto-β-BA (KBA **2**), β-BA **3**, and 3-*O*-acteyl-β-BA (ABA **4**) are pentacyclic triterpene acids that represent major ingredients in lipophilic frankincense extracts, reaching 14 to 25% (m/m) [7,10]. Previous studies have shown that BAs display potent anti-inflammatory properties by interfering with multiple targets that are involved in the initiation and maintenance of inflammation [6,11]. For example, BAs were reported to inhibit the key enzymes 5-LO [12,13], COX [14], and microsomal prostaglandin E_2_ synthase (mPGES)-1 [15] in pro-inflammatory LT and prostaglandin (PG)E_2_ biosynthesis, the neutrophil proteases elastase [16] and cathepsin G [17], the immunoregulating antimicrobial peptide LL-37 [18], inhibitor of NFκB (IkB) kinases [19] and lipopolysaccharide (LPS) activity [20]. Inhibition of 5-LO, mPGES-1 and cathepsin G received much attention as major targets of BAs being responsible for suppressing inflammation, and animal studies as well as clinical trials confirmed that isolated BAs or frankincense extracts suppress PGE_2_ formation and cathepsin G activity in vivo [15,17,21]. Notably, the potencies of the four different BAs varied depending on the nature of the target, and in many cases (e.g., for 5-LO, cathepsin G, and mPGES-1), AKBA was the most potent derivative [11,12,13,15,17].

We previously showed that for mPGES-1, other triterpene acids from frankincense than BAs inhibit the enzyme with even superior potencies versus BAs [9]. Thus, the tetracyclic tirucallic acids **14**–**17** and the pentacyclic lupeolic acid derivative **12** were found to be up to 8-fold more potent than AKBA in vitro [9], implying that these triterpene acids may contribute to the pharmacological/anti-inflammatory properties of crude frankincense extracts as well. Since only BAs have been evaluated for inhibition of 5-LO and cathepsin G, we here performed a comprehensive analysis of frankincense-derived tetra- and pentacyclic triterpene acids that we investigated for interference with 5-LO and cathepsin G. Moreover, a panel of novel semi-synthetic BA-derivatives were synthesized and examined along these lines, to further explore the critical pharmacophores.

## 2. Results

### 2.1. Isolation and Semi-Synthesis of the Triterpene Acids

The four BAs **1**–**4**, the three roburic acids **5**–**7**, dihydro-nyctanthic acid (DHNA **8**), the four lupeolic acids **9**–**12**, and the five tirucallic acids **13**–**17** (Table 1) were isolated from gum resins of different *Boswellia* species by preparative high-performance liquid chromatography (HPLC), as described previously [9,22]. The *R*- and *S*-configured ally-alcohols **18** and **19** were synthesized from KBA and AKBA using a combination of LiBr and NaBH_4_ in diglyme at reflux temperature, as described [23,24]. The ABA and AKBA derivatives with modified C3 position were prepared as follows: β-BA **3** or KBA **2** were treated with oxalyl chloride to form the half-ester oxaloyl-BA **20** and oxaloyl-KBA **21**; β-BA **3** or KBA **2** were treated with succinic or glutaric anhydride to form the half-ester succinoyl-BA **22** and succinoyl-KBA **23** as well as glutaroyl-BA **24** and glutaroyl-KBA **25**; the carboxymethyl-BA **26** and carboxymethyl-KBA **27** were synthesized from β-BA **3** or KBA **2** using 2-chloro acetic acid and NaH in THF over night as described earlier [23,24]. All compounds were analyzed by ^1^H- and ^13^C-NMR, as well as by mass spectrometry.

### 2.2. Inhibition of 5-LO by Natural Occurring Triterpene Acids

In analogy to our previous SAR study on 17 triterpene acids from frankincense that were tested for inhibition of mPGES-1 activity in a cell-free assay [9], we analyzed the effects of these compounds for their inhibitory effects against human isolated 5-LO in a suitable cell-free test system, where 20 µM arachidonic acid was exogenously added as substrate [25]. The well-recognized 5-LO inhibitor BWA4C [25] was used as reference drug that inhibited 5-LO with IC_50_ = 0.04 µM. In agreement with previous data [13], AKBA **1** and KBA **2** efficiently inhibited 5-LO activity with IC_50_ = 3.0 and 4.6 µM, respectively, while β-BA **3** and ABA **4** suppressed 5-LO activity at 10 µM by only 21–26% (Table 1). Among the three roburic acids **5**–**7**, only **7** caused some (27%) inhibition at 10 µM, while **5** and **6** failed in this respect. Similarly, DHNA **8** and the lupeolic acids **9** and **10** did not markedly inhibit 5-LO, but the hydroxylated lupeolic acids **11** and **12** were significantly active, with IC_50_ = 10 and 8.3 µM, respectively (Table 1). Among the tirucallic acids (**13**–**17**), **14** and **15** caused weak 5-LO inhibition at 10 µM (34 and 36%, respectively), and other members were hardly active. Together, AKBA **1** and KBA **2** are clearly the most potent direct 5-LO inhibitors out of the 17 triterpene acids from frankincense, which underlines that different structural requirements of triterpene acids are required for efficient inhibition of 5-LO versus mPGES-1 [9].

Several studies reported that frankincense extracts, as well as AKBA **1** and KBA **2**, are superior inhibitors of 5-LO in intact cells as compared to cell-based assays, for multiple possible reasons that remain unclear [6,12,13,26]. Thus, we studied the triterpene acids also against 5-LO activity in a well-established cell-based assay using human neutrophils stimulated with A23187 [25]. The reference 5-LO inhibitor BWA4C was about 4-fold less active in neutrophils (IC_50_ = 0.16 µM) as compared to cell-free assay conditions. Both AKBA **1** and KBA **2** were somewhat more potent in intact cells (IC_50_ = 3.0 and 3.5 µM) versus isolated 5-LO, and also for β-BA **3**, but not for ABA **4**, where slightly more efficient 5-LO inhibition was observed (47% inhibition at 10 µM, Table 1). Again, roburic acids **5** and **6** as well as DHNA **8** were hardly active, but **7** caused potent suppression of 5-LO with IC_50_ = 4.3 µM, being almost equally effective as AKBA **1** and KBA **2**. Along these lines, also, the lupeolic acids **9**, **11**, and **12** (but not **10**) suppressed 5-LO product formation, with IC_50_ = 4.0, 4.6, and 5.1 µM. Although tirucallic acids **13**–**17** failed to inhibit isolated 5-LO, **13**–**16** were quite effective in intact cells, with **14** being about 3-fold more potent (IC_50_ = 1.1 µM) than AKBA **1**, and with **13** and **15** displaying similar potencies (IC_50_ = 2.9 and 3 µM). In conclusion, among the 17 triterpene acids, the tirucallic acid **14** is the most potent inhibitor of 5-LO product biosynthesis in neutrophils, thus outperforming AKBA, despite lack of pronounced effectiveness against isolated 5-LO.

### 2.3. Inhibition of Cathepsin G by Natural Occurring Triterpene Acids

Since cathepsin G is a pharmacological relevant target for BAs and frankincense with high affinities to BAs [17], we analyzed also the effects of the 17 triterpene acids for inhibition of cathepsin G activity in a cell-free assay. Since cathepsin G is a secreted protease that acts outside the cell upon degranulation, cell-based assays are not feasible for cathepsin G inhibitor studies as compared to 5-LO. JNJ-1031795 was used as reference drug [27] that blocked cathepsin G activity with IC_50_ = 0.06 µM. The rank order for cathepsin G inhibition by the BAs was β-BA **3** > AKBA **1** > ABA **4** > KBA **2**, with IC_50_ = 0.5, 0.6, 2.0, and 4.1 µM, respectively. Among all other triterpene acids tested at 10 µM, only the lupeolic acid **10** exhibited >50% inhibition of cathepsin G with an IC_50_ = 7.5 µM. Nevertheless, marked suppression of cathepsin G activity (i.e., 27–44% inhibition at 10 µM) was found for all roburic acids (**5**–**7**), DHNA **8**, lupeolic acids **9** and **11**, and tirucallic acid **13**, **14**, **16**, and **17**. Only **12** and **15** failed to markedly inhibit cathepsin G. Together, cathepsin G is a clearly preferred target for BAs (particularly β-BA **3** and AKBA **1**), with much less susceptibility for all other triterpene acids tested.

### 2.4. Effects of Semi-Synthetic BAs against 5-LO and Cathepsin G

Next, we investigated a set of semi-synthetic BA derivatives for inhibition of 5-LO and cathepsin G to further explore the structure-activity relationships (SARs) of BAs. First, the 11-keto moiety of KBA was reduced to hydroxy, yielding the *R*- and *S*-configured ally-alcohols **18** and **19**. Of interest, **18** was somewhat more efficient to inhibit 5-LO in neutrophils (IC_50_ = 2.8 µM) versus parental KBA **2** while **19** was less active, and both compounds exhibited only weak effects against 5-LO in cell-free assays (<25% inhibition at 10 µM). Also, cathepsin G activity was less pronouncedly affected by **18** and **19**. Then, we replaced the 3-*O*-acetyl moiety of AKBA **1** or ABA **4** by acidic moieties, such as oxaloyl, succinoyl, glutaroyl, and carboxymethyl groups. All these compounds were not or only weak 5-LO inhibitors, with <25% inhibition at 10 µM in cell-free assays. Similarly, in intact cells, 5-LO product formation was not or barely affected (IC_50_ > 10 µM); the most potent compound was the carboxymethyl-BA **26** that inhibited 5-LO activity by 42% at 10 µM. By contrast, pronounced cathepsin G inhibition was observed for BA derivatives lacking the 11-keto moiety, such as oxaloyl-BA **20**, glutaroyl-BA **24**, and carboxymethyl-BA **26**, with IC_50_ = 4.7, 3.6, and 3.4 µM. Of interest, even though AKBA **1** (an 11-keto derivative carrying a 3-acetoyl moiety) was quite potent (IC_50_ = 0.6 µM), the 11-keto derivatives oxaloyl-KBA **21**, succinoyl-KBA **23**, and carboxymethyl-KBA **27** were not active, and glutaroyl-KBA **25** (IC_50_ = 8.6) was less efficient than the respective counterpart, **24**, lacking the 11-keto moiety. Taken together, modification of the acetoyl group of AKBA **1** or ABA **4** towards acidic residues is strongly detrimental for inhibition of 5-LO, and to a varying extent, also for interference with cathepsin G, depending on the presence of the 11-keto moiety.

## 3. Discussion

Here, we report the SAR of various natural occurring triterpene acids from frankincense, and of novel semi-synthetic BA derivatives for inhibition of the two molecular targets, 5-LO and cathepsin G. For 5-LO, the SARs depend on the assay type: for direct interaction with 5-LO in the cell-free assay, AKBA **1** and KBA **2** were most potent, and neither natural nor semi-synthetic triterpene acids were comparably active. Modification of the acetoyl group of AKBA **1** towards acidic residues is clearly detrimental for interference with 5-LO. For inhibition of 5-LO product formation in neutrophils, however, several of the natural triterpene acids were efficient and outperformed AKBA **1**, such as the tirucallic acids **13** and **14**. Since tirucallic acids were shown to interfere with various signaling pathways [28,29], among others, Ca^2+^ mobilization and mitogen-activated protein kinase kinase (MEK)-1/2, that could also activate 5-LO in intact cells, one may speculate that tirucallic acids and possibly also roburic and lupeolic acids interfere with the activation of 5-LO, and thus, with 5-LO product biosynthesis. In fact, lupeolic acids (e.g., **12**) were shown to suppress the biosynthesis of various eicosanoids by interference with cytosolic phospholipase A2 [8] that provides substrate to 5-LO.

In analogy to interference with 5-LO, inhibition of cathepsin G activity was also most efficient for BAs, particularly for β-BA **3** and AKBA **1**, with submicromolar IC_50_ values. Among the natural triterpene acids, only the lupeolic acid **10** reached an IC_50_ below 10 µM, even though several other triterpene acids significantly inhibited cathepsin G at 10 µM, albeit less than 50%. Among the semisynthetic BAs lacking the 11-keto moiety, oxaloyl-BA **20**, glutaroyl-BA **24**, and carboxymethyl-BA **26** were efficient cathepsin G inhibitors, indicating that the 11-keto moiety is dispensable for cathepsin G interference. 

In summary, among the 17 investigated natural occurring triterpene acids from frankincense, the BAs provide the most potent inhibitors of 5-LO (i.e., AKBA **1**) and cathepsin G (i.e., β-BA **3**). Nevertheless, some lupeolic acids are efficient as well, and for inhibition of 5-LO product formation in intact cells, diverse representatives from all types of triterpene acids are active, even with superior potency (e.g., tirucallic acid **14**) than BAs. Notably, our previous SAR study on the same 17 triterpene acids for inhibition of mPGES-1 revealed opposite findings: thus, all six tirucallic acids were more potent than the BAs, and also, the lupeolic acid **12** and the roburic acid **7** were clearly superior [9]. Together, dual interference with cathepsin G and 5-LO product formation in human neutrophils, on top of mPGES-1 inhibition, represents a beneficial pharmacological profile, and may qualify triterpene acids as anti-inflammatory natural products and pharmacological leads for intervention with inflammatory diseases.

## 4. Materials and Methods 

### 4.1. Chemistry

General information. All reactions were run in dried solvents under N_2_ in flame dried glassware. THF and diethyl ether were dried with Na/benzophenone under N_2_. Flash chromatography was done with silica gel (Merck, Darmstadt, Germany) with particle size 40–63 µm and with distilled solvents. Reagents were bought from chemical suppliers and used without further purification.

NMR spectra (^1^H, ^13^C, Distortionless Enhancement of Polarization Transfer (DEPT), H,H-Correlation Spectroscopy (H,H-COSY), Hetero Single Quantum Coherence (HSQC), Hetero Multiple Bond Correelation (HMBC), Nuclear Overhauser Effect Spectroscopy (NOESY) were recorded with a Bruker AV 500 (Bruker, Rheinstetten, Germany) at room temperature (rt). Mass spectra (MS, HRMS) were recorded on a Finnigan MAT 95S (Thermo Fisher Scientific, Waltham, MA, USA). Optical rotations were measured on a Perkin Elmer polarimeter type 241MC (Perkin Elmer, Rodgau, Germany) 

Melting points were measured with a Büchi melting point apparatus (Dr. Tottolli) (Büchi Labortechnik GmbH, Essen, Germany) and are uncorrected. The following original data on the chemistry of compounds **18**–**27** are reported also in the doctoral thesis by Nicole Kather, 2007 [24].

11α-Hydroxy-β-boswellic acid **18** and 11β-hydroxy-β-boswellic acid **19**. NaBH_4_ (121 mg, 3.2 mmol) was dissolved in dry diglyme (3.5 mL). LiBr (278 mg, 3.2 mmol) was added with stirring at rt. After 30 min, β-KBA **2** (300 mg, 0.64 mmol) was added and the mixture was refluxed for 1 h. After cooling to rt, the mixture was diluted with cold water (approx. 5 mL) and acidified carefully to pH 4 with 1 N HCl. The mixture was extracted three times with diethyl ether (ca. 10 mL each). The combined ethereal extracts were washed with water and brine, dried with MgSO_4_, and filtered, and the solvent was evaporated in vacuo at rt. The crude product was purified by flash chromatography (pentane/diethyl ether 1:1 + 1% acetic acid) to yield 83 mg of **18** and 141 mg of **19** as white solids. 

11-α-Hydroxy-β-boswellic acid **18**: ^1^H-NMR ((CD_3_)_2_CO, 500.13 MHz): δ [ppm] = 5.22 (d, *J =* 2.1 Hz, 1H, H-12), 4.21 (d, *J =* 9.1 Hz, 1H, H-11), 3.98 (bs, 1H, H-3), 2.22–2.06 (m, 3H, H-1β, H-2β, H-16α), 1.94–1.86 (m, 1H, H-6β), 1.84–1.75 (m, 1H, H-15β), 1.75–1.68 (m, 2H, H-6α, H-9), 1.68–1.60 (m, 2H, H-1α, H-5), 1.60–1.50 (m, 1H, H-7α), 1.50–1.30 (m, 8H, H-2α, H-7β, H-18, H-19, H-21α, H-21β, H-22α, H-22β), 1.29 (s, 3H, H-23), 1.22 (bs, 4H, H-15α, H-27), 1.14 (s, 3H, H-26), 1.09 (s, 3H, H-25), 0.93 (bs, 5H, H-16β, H-20, H-30), 0.90 (d, *J =* 6.2 Hz, 3H, H-29), 0.83 (s, 3H, H-28).

^13^C-NMR ((CD_3_)_2_CO, 125.76 MHz): δ [ppm] = 180.3 (C-24, COOH), 142.2 (C-13, H>C=C<), 132.8 (C-12, H>C=C<), 71.7 (C-3, HO>CH-), 69.6 (C-11, HO>CH-), 60.3 (C-18, >CH-), 55.4 (C-9, >CH-), 50.7 (C-5, >CH–), 49.3 (C-4, >C<), 45.0 (C-8, >C<), 44.1 (C-14, >C<), 43.2 (C-22, –CH2–), 41.4 (C-19, >CH–), 41.4 (C-20, >CH–), 40.5 (C-10, >C<), 37.8 (C-1, –CH_2_–), 36.1 (C-7, –CH_2_–), 35.5 (C-17, >C<), 32.9 (C-21, –CH_2_–), 30.2 (C-28, –CH_3_), 29.8 (C-16, –CH_2_–), 28.4 (C-15, –CH_2_–), 28.3 (C-2, –CH_2_–), 26.2 (C-23, –CH_3_), 24.3 (C-27, –CH_3_), 22.6 (C-30, –CH_3_), 21.6 (C-6, –CH_2_–), 19.7 (C-26, –CH_3_), 18.9 (C-29, –CH_3_), 15.8 (C-25, –CH_3_). 

MS (EI, 70 eV): *m*/*z* (%) = 454 (100) [M-H_2_O]^+^, 439 (8), 421 (12), 325 (4), 301 (12), 269 (8), 255 (45), 253 (6), 237 (4), 215 (5). HRMS (EI, 70 eV): calculated: 472.3587 for C_30_H_48_O_4_; found: 472.3570. [α]^D^: +51.7° (c = 1.61, acetone). m.p. 166–169 °C (dec).

11-β-Hydroxy-β-boswellic acid **19**: ^1^H-NMR ((CD_3_)_2_CO, 500.13 MHz): δ [ppm] = 5.30 (d, *J =* 4.3 Hz, 1H, H-12), 4.45 (t, *J =* 4.7 Hz, 1H, H-11), 4.00 (t, *J =* 2.4 Hz, 1H, H-3), 2.33–2.25 (m, 1H, H-2β), 2.12–2.05 (m, 1H, H-16α), 2.00–1.88 (m, 2H, H-6β, H-15β), 1.84–1.78 (m, 1H, H-1β), 1.76–1.70 (m, 1H, H-6α), 1.70–1.60 (m, 2H, H-1α, H-7α), 1.58–1.50 (m, 3H, H-2α, H-5, H-9), 1.47–1.43 (m, 1H, H-22α), 1.43 (s, 3H, H-25), 1.43–1.36 (m, 3H, H-18, H-19, H-21β), 1.36–1.33 (m, 3H, H-7β, H-21α, H-22β), 1.33 (s, 3H, H-26), 1.28 (s, 3H, H-23), 1.15–1.10 (m, 1H, H-15α), 1.09 (s, 3H, H-27), 1.09–1.06 (m, 1H, H-16β), 0.93 (s, 3H, H-30), 0.93–0.87 (m, 1H, H-20), 0.87 (s, 3H, H-28), 0.84 (d, *J =* 5.9 Hz, 3H, H-29).

^13^C-NMR ((CD_3_)_2_CO, 125.76 MHz): δ [ppm] = 180.2 (C-24, COOH), 142.1 (C-13, H>C=C<), 131.7 (C-12, H>C=C<), 71.8 (C-3, HO>CH–), 66.8 (C-11, HO>CH–), 60.8 (C-18, >CH–), 53.9 (C-9, >CH–), 51.7 (C-5, >CH–), 48.7 (C-4, >C<), 44.4 (C-14, >C<), 43.3 (C-22, –CH_2_–), 41.6 (C-8, >C<), 41.4 (C-19, >CH–), 41.4 (C-20, >CH–), 40.6 (C-10, >C<), 35.6 (C-1, –CH_2_–), 35.4 (C-17, >C<), 35.4 (C-7, –CH_2_–), 32.9 (C-21, –CH_2_–), 30.4 (C-28, –CH_3_), 29.8 (C-16, –CH_2_–), 29.0 (C-15, –CH_2_–), 28.1 (C-2, –CH_2_–), 25.8 (C-23, –CH_3_), 23.9 (C-27, –CH_3_), 22.3 (C-30, –CH_3_), 22.1 (C-6, –CH_2_–), 20.7 (C-26, –CH_3_), 18.7 (C-29, –CH_3_), 17.7 (C-25, –CH_3_).

MS (EI, 70 eV): *m*/*z* (%) = 472 (4) [M]^+^, 454 (100), 421 (8), 273 (2), 255 (6), 234 (3), 203 (3). HRMS (EI, 70 eV): calculated: 472.3552 for C_30_H_48_O_4_; found: 472.3552. [α]^D^: +117.5° (c = 0.63, acetone). m.p. 125–128 °C (dec).

3*-O-*Oxaloyl-β-boswellic acid **20**. β-BA **3** (150 mg, 0.33 mmol) was dissolved in dry THF (2.3 mL). This solution was added dropwise with stirring to a solution of oxaly dichloride (288 μL, 3.30 mmol) in THF (1 mL). After 30 min at rt, cold water (ca. 50 mL) was added, and the mixture was extracted three times with diethyl ether (ca. 10 mL each). The combined ethereal extracts were washed with H_2_O and brine, and dried with MgSO_4_. After filtering, the MgSO_4_ the solvent was evaporated in vacuo to yield 140 mg of **20**.

^1^H-NMR ((CD_3_)_2_CO, 500.13 MHz): δ [ppm] = 5.40 (t, *J =* 2.6 Hz, 1H, H-3), 5.20 (t, *J =* 3.5 Hz, 1H, H-12), 2.31–2.22 (m, 1H, H-2β), 2.12–2.08 (m, 1H, H-16α), 2.01–1.85 (m, 4H, H-6β, H-11α, H-11β, H-15β), 1.82–1.76 (m, 1H, H-6α), 1.75–1.65 (m, 2H, H-2α, H-9), 1.65–1.53 (m, 3H, H-1β, H-5, H-7α), 1.48–1.28 (m, 7H, H-1α, H-7β, H-18, H-19, H-21β, H-22α, H-22β), 1.28 (bs, 4H, H-21α, H-23), 1.15 (s, 3H, H-27), 1.10 (s, 3H, H-26), 1.08–1.04 (m, 1H, H-15α), 0.99 (s, 3H, H-25), 0.93 (bs, 4H, H-20, H-30), 0.83 (s, 3H, H-28), 0.83 (t, *J =* 6.0 Hz, 3H, H-29).

^13^C-NMR ((CD_3_)_2_CO, 125.76 MHz): δ [ppm] = 178.5 (C-24, COOH), 160.3 (C-32, COOH (oxalyl)), 160.0 (C-31, >C=O (oxalyl)), 141.4 (C-13, H>C=C<), 126.5 (C-12, H>C=C<), 78.5 (C-3, HO>CH–), 61.0 (C-18, >CH–), 52.1 (C-5, >CH–), 48.6 (C-9, >CH–),48.2 (C-4, >C<), 44.0 (C-8, >C<), 43.2 (C-22, –CH_2_–), 41.8 (C-14, >C<), 41.5 (C-19, >CH–), 41.4 (C-20, >CH–), 39.1 (C-10, >C<), 36.2 (C-1, –CH_2_–), 35.5 (C-17, >C<), 34.8 (C-7, –CH_2_–), 32.9 (C-21, –CH_2_–), 30.2 (C-28, –CH_3_), 29.8 (C-16, –CH_2_–), 28.2 (C-15, –CH_2_–), 25.1 (C-2, C-11, –CH_2_–), 25.0 (C-23, –CH_3_), 24.7 (C-27, –CH_3_), 22.6 (C-30, –CH_3_), 21.5 (C-6, –CH_2_–), 18.9 (C-29, –CH_3_), 18.4 (C-26, –CH_3_), 14.8 (C-25, –CH_3_).

MS (CI, 150 eV): *m*/*z* (%) = 484 (5) [M-CO_2_]^+^, 447 (12), 394 (7), 307 (14), 231 (4), 218 (100), 203 (17). HRMS (CI, 150 eV): calculated: 484.3495 for C_31_H_48_O_4_ (C_32_H_48_O_6_-CO_2_); found: 484.3524. [α]^D^: +56.5° (c = 0.78, acetone). m.p. 212–215 °C (dec).

3-*O*-Oxaloyl-11-keto-β-boswellic acid **21** was prepared analogously to **20** (see above).

^1^H-NMR (CDCl_3_, 500.13 MHz): δ [ppm] = 5.57 (s, 1H, H-12), 5.43 (bs, 1H, H-3), 2.61–2.56 (m, 1H, H-1β), 2.46 (s, 1H, H-9), 2.35–2.25 (m, 1H, H-2β), 2.15–2.05 (m, 1H, H-16α), 1.96–1.84 (m, 2H, H-6β, H-15β), 1.80–1.65 (m, 3H, H-2α, H-6α, H-7α), 1.57–1.37 (m, 6H, H-5, H-7β, H-18, H-19, H-21β, H-22α), 1.34 (s, 3H, H-27), 1.30 (s, 3H, H-23), 1.30–1.24 (m, 3H, H-1α, H-21α, H-22β), 1.19 (bs, 4H, H-15α, H-26), 1.16 (s, 3H, H-25), 1.05–0.98 (m, 1H, H-16β), 0.94 (bs, 4H, H-20, H-30), 0.82 (s, 3H, H-28), 0.79 (d, *J =* 6.3 Hz, 3H, H-29).

^13^C-NMR (CDCl_3_, 125.76 MHz): δ [ppm] = 199.7 (C-11, >C=O), 180.8 (C-24, COOH), 165.9 (C-13, H>C=C<), 158.8 (C-32, COOH (oxalyl)), 157.8 (C-31, >C=O (oxalyl)), 130.2 (C-12, H>C=C<), 77.3 (C-3, HO>CH–), 60.1 (C-9, >CH–), 59.1 (C-18, >CH–), 50.1 (C-5, >CH–), 46.6 (C-4, >C<), 45.1 (C-8, >C<), 43.8 (C-14, >C<), 40.9 (C-22, –CH_2_–), 39.3 (C-19, >CH–), 39.3 (C-20, >CH–), 37.3 (C-10, >C<), 34.2 (C-1, –CH_2_–), 34.0 (C-17, >C<), 32.7 (C-7, –CH_2_–), 30.9 (C-21, –CH_2_–), 28.9 (C-28, –CH_3_), 27.5 (C-16, –CH_2_–), 27.2 (C-15, –CH_2_–), 23.9 (C-23, –CH_3_), 23.3 (C-2, –CH_2_–), 21.1 (C-30, –CH_3_), 20.5 (C-27, –CH_3_), 18.7 (C-6, –CH_2_–), 18.4 (C-26, –CH_3_), 17.4 (C-29, –CH_3_), 13.3 (C-25, –CH_3_).

MS (CI, 150 eV): *m*/*z* (%) = 499 (100) [M-CO_2_ + H]^+^, 453 (42), 409 (44), 408 (16), 393 (6), 299 (5), 287 (3), 273 (90), 232 (91). HRMS (CI, 150 eV): calculated: 498,3395 for C_31_H_46_O_5_ (C_32_H_46_O_7_-CO_2_); found: 498.3370. [α]^D^: +67.0° (c = 0.98, acetone). m.p. 188–191 °C (dec).

3-*O*-Succinoyl-β-boswellic acid **22**. β-BA **3** was dissolved in dry pyridine (2.2 mL). Succinic anhydride (220 mg, 2.2 mmol) was added, followed by 4-pyrrolidino pyridine (36.2 mg, 0.22 mmol). The reaction mixture was refluxed for 7 h. After cooling to rt, the dark brown mixture was diluted with diethyl ether (ca. 20 mL) and was washed three times with 1 N HCl (ca. 10 mL each). The organic phase was washed with H_2_O and brine, and dried with MgSO_4_. After filtration, the solvent was removed in vacuo to yield an orange-brown solid which was purified by flash chromatography (pentane/diethyl ether 2:1 + 1% HOAc) to give 87 mg of **22** as a white solid.

^1^H-NMR (CDCl_3_, 500.13 MHz): δ [ppm] = 5.36 (t, *J =* 2.3 Hz, 1H, H-3), 5.16 (t, *J =* 3.4 Hz, 1H, H-12), 2.78–2.62 (m, 4H, H-32, H-33 (Succinyl)), 2.17–2.10 (m, 1H, H-2β), 2.05–1.99 (m, 1H, H-16α), 1.95–1.90 (m, 2H, H-11α, H-11β), 1.88–1.77 (m, 2H, H-6β, H-15β), 1.72–1.67 (m, 1H, H-6α), 1.64–1.56 (m, 2H, H-2α, H-9), 1.56–1.47 (m, 2H, H-1β, H-7α), 1.47–1.36 (m, 4H, H-5, H-7β, H-21β, H-22α), 1.36–1.30 (m, 2H, H-18, H-19), 1.30–1.22 (m, 2H, H-21α, H-22β), 1.22 (bs, 4H, H-1α, H-23), 1.12 (s, 3H, H-27), 1.05 (s, 3H, H-26), 1.05–0.98 (m, 1H, H-15α), 0.94–0.86 (m, 8H, H-16β, H-20, H-25, H-30), 0.81 (s, 3H, H-28), 0.81 (bs, 3H, H-29).

^13^C-NMR (CDCl_3_, 125.76 MHz): δ [ppm] = 182.3 (C-24, COOH), 177.9 (C-34, COOH (succinyl)), 171.1 (C-31, >C=O (succinyl)), 139.6 (C-13, H>C=C<), 124.5 (C-12, H>C=C<), 73.8 (C-3, HO>CH–), 59.2 (C-18, >CH–), 50.6 (C-5, >CH–), 46.8 (C-9, >CH–), 46.8 (C-4, >C<), 42.3 (C-14, >C<), 41.6 (C-22, –CH_2_–), 40.1 (C-8, >C<), 39.8 (C-19, >CH–), 39.6 (C-20, >CH–), 37.4 (C-10, >C<), 34.6 (C-1, –CH_2_–), 33.8 (C-17, >C<), 33.1 (C-7, –CH_2_–), 31.3 (C-21, –CH_2_–), 29.3 (C-32*/C-33*, –CH_2_– (succinyl)), 29.0 (C-32*/C-33*, –CH_2_– (succinyl)), 28.8 (C-28, –CH_3_), 28.2 (C-16, –CH_2_–), 26.6 (C-15, –CH_2_–), 23.7 (C-23, –CH_3_), 23.7 (C-2, –CH_2_–), 23.4 (C-11, –CH_2_–), 23.2 (C-27, –CH_3_), 21.4 (C-30, –CH_3_), 19.6 (C-6, –CH_2_–), 17.5 (C-29, –CH_3_), 16.9 (C-26, –CH_3_), 13.4 (C-25, –CH_3_); *ambiguous.

MS (CI, 150 eV): *m*/*z* (%) = 556 (8) [M]^+^, 439 (32), 394 (13), 379 (4), 231 (6), 218 (100), 203 (10). HRMS (CI, 150 eV): calculated: 556.3780 for C_34_H_52_O_6_; found: 556.3772. [α]^D^: +57.0° (c = 2.02, chloroform). m.p. 176–179 °C (dec).

3*-O-*Succinoyl-11-keto-β-boswellic acid **23** was prepared analogously to **22**.

^1^H-NMR (CDCl_3_, 500.13 MHz): δ [ppm] = 5.57 (s, 1H, H-12), 5.35 (bs, 1H, H-3), 2.75–2.63 (m, 4H, H-32, H-33 (succinyl)), 2.57–2.52 (m, 1H, H-1β), 2.43 (s, 1H, H-9), 2.25–2.05 (m, 2H, H-2β, H-16α), 1.95–1.85 (m, 2H, H-6β, H-15β), 1.75–1.65 (m, 2H, H-6α, H-7α), 1.61–1.36 (m, 7H, H-2α, H-5, H-7β, H-18, H-19, H-21β, H-22α), 1.35 (s, 3H, H-27), 1.35–1.30 (m, 2H, H-21α, H-22β), 1.22 (bs, 4H, H-15α, H-23), 1.19 (bs, 4H, H-1α, H-26), 1.14 (s, 3H, H-25), 1.04–0.98 (m, 1H, H-16β), 0.95 (bs, 4H, H-20, H-30), 0.83 (s, 3H, H-28), 0.82 (d, *J =* 6.5 Hz, 3H, H-29).

^13^C-NMR (CDCl_3_, 125.76 MHz): δ [ppm] = 199.4 (C-11, >C=O), 181.7 (C-24, COOH), 177.8 (C-34, COOH (succinyl)), 170.8 (C-31, >C=O (succinyl)), 165.1 (C-13, H>C=C<), 130.5 (C-12, H>C=C<), 73.6 (C-3, HO>CH–), 60.3 (C-9, >CH–), 59.1 (C-18, >CH–), 50.4 (C-5, >CH–), 46.6 (C-4, >C<), 45.1 (C-8, >C<), 43.8 (C-14, >C<), 40.9 (C-22, –CH_2_–), 39.3 (C-19, >CH–), 39.3 (C-20, >CH–), 37.4 (C-10, >C<), 34.6 (C-1, –CH_2_–), 34.0 (C-17, >C<), 32.9 (C-7, –CH_2_–), 30.9 (C-21, –CH_2_–), 29.4 (C-32*/C-33*, –CH_2_– (succinyl)), 29.1 (C-32*/C-33*, –CH_2_– (succinyl)), 28.9 (C-28, –CH_3_), 27.6 (C-16, –CH_2_–), 27.3 (C-15, –CH_2_–), 23.8 (C-23, –CH_3_), 23.6 (C-2, –CH_2_–), 21.1 (C-30, –CH_3_), 20.5 (C-27, –CH_3_), 18.7 (C-6, –CH_2_–), 18.4 (C-26, –CH_3_), 17.4 (C-29, –CH_3_), 13.3 (C-25, –CH_3_).

MS (CI, 150 eV): *m*/*z* (%) = 571 (13) [M + H]^+^, 471 (4), 453 (17), 409 (100), 408 (38), 393 (7), 379 (15), 273 (36), 232 (19), 218 (10). HRMS (CI, 150 eV): calculated: 570.3666 for C_34_H_50_O_7_; found: 570.3611. [α]^D^: +79.7° (c = 1.64, chloroform). m.p. 169–172 °C (dec).

3-*O*-Glutaroyl-β-boswellic acid **24** was prepared analogously to **22**.

^1^H-NMR ((CD_3_)_2_CO, 500.13 MHz): δ [ppm] = 5.30 (t, *J =* 2.4 Hz, 1H, H-3), 5.20 (t, *J =* 3.4 Hz, 1H, H-12), 2.46 (t, *J =* 7.4 Hz, 2H, H-34 (glutaroyl)), 2.40 (t, *J =* 7.4 Hz, 2H, H-32 (glutaroyl)), 2.21–2.14 (m, 1H, H-2β), 2.11–2.07 (m, 1H, H-16α), 2.00–1.86 (m, 6H, H-6β, H-11α, H-11β, H-15β, H-33 (glutaroyl)), 1.78–1.75 (m, 1H, H-6α), 1.70–1.67 (m, 1H, H-9), 1.64–1.58 (m, 2H, H-2α, H-7α), 1.55–1.49 (m, 2H, H-1β, H-5), 1.47–1.26 (m, 8H, H-1α, H-7β, H-18, H-19, H-21α, H-21β, H-22α, H-22β), 1.23 (s, 3H, H-23), 1.15 (s, 3H, H-27), 1.09 (s, 3H, H-26), 1.07–1.02 (m, 1H, H-15α), 0.98 (s, 3H, H-25), 0.94-0.89 (m, 5H, H-16β, H-20, H-30), 0.84 (s, 3H, H-28), 0.84 (d, *J =* 5.8 Hz, 3H, H-29).

^13^C-NMR ((CD_3_)_2_CO, 125.76 MHz): δ [ppm] = 179.0 (C-24, COOH), 175.1 (C-35, COOH (glutaroyl)), 173.6 (C-31, >C=O (glutaroyl)), 141.4 (C-13, H>C=C<), 126.6 (C-12, H>C=C<), 75.0 (C-3, HO>CH–), 61.1 (C-18, >CH–), 52.3 (C-5, >CH–), 48.7 (C-9, >CH–), 48.2 (C-4, >C<), 44.0 (C-8, >C<), 43.3 (C-22, –CH_2_–), 41.9 (C-14, >C<), 41.5 (C-19, >CH–), 41.4 (C-20, >CH–), 39.1 (C-10, >C<), 36.5 (C-1, –CH_2_–), 35.5 (C-17, >C<), 35.1 (C-34, –CH_2_– (glutaroyl)), 34.9 (C-7, –CH_2_–), 34.3 (C-32, –CH_2_– (glutaroyl)), 32.9 (C-21, –CH_2_–), 30.3 (C-28, –CH_3_), 29.8 (C-16, –CH_2_–), 28.3 (C-15, –CH_2_–), 25.2 (C-23, –CH_3_), 25.4 (C-2, –CH_2_–), 25.1 (C-11, –CH_2_–), 24.7 (C-27, –CH_3_), 22.7 (C-30, –CH_3_), 22.1 (C-33, –CH_2_– (glutaroyl)), 21.6 (C-6, –CH_2_–), 18.9 (C-29, –CH_3_), 18.4 (C-26, –CH_3_), 14.9 (C-25, –CH_3_).

MS (EI, 70 eV): *m*/*z* (%) = 570 (8) [M]^+^, 471 (8), 454 (12), 438 (28), 423 (19), 394 (25), 379 (15), 218 (100), 203 (33), 189 (22). HRMS (EI, 70 eV): calculated: 570.3982 for C35H54O6; found: 570.3951. [α]^D^: +56.7° (c = 0.72, acetone). m.p. 137–140 °C (dec).

3-*O*-Glutaroyl-11-keto-β-boswellic acid **25** was prepared analogously to **22**.

^1^H-NMR ((CD_3_)_2_CO, 500.13 MHz): δ [ppm] = 5.49 (s, 1H, H-12), 5.29 (t, *J =* 2.6 Hz, 1H, H-3), 2.53–2.48 (m, 2H, H-1β, H-9), 2.44 (t, *J =* 7.4 Hz, 2H, H-34 (glutaroyl)), 2.39 (t, *J =* 7.4 Hz, 2H, H-32 (glutaroyl)), 2.27–2.16 (m, 2H, H-2β, H-16α), 1.97–1.88 (m, 4H, H-6β, H-15β, H-33 (glutaroyl)), 1.80–1.74 (m, 2H, H-6α, H-7α), 1.61–1.43 (m, 7H, H-2α, H-5, H-7β, H-18, H-19, H-21β, H-22α), 1.39 (s, 3H, H-27), 1.39–1.35 (m, 2H, H-21α, H-22β), 1.33–1.25 (m, 2H, H-1α, H-15α), 1.23 (s, 3H, H-23), 1.21 (s, 3H, H-26), 1.18 (s, 3H, H-25), 1.07–1.02 (m, 1H, H-16β), 0.96 (bs, 4H, H-20, H-30), 0.86 (s, 3H, H-28), 0.83 (d, *J =* 6.4 Hz, 3H, H-29).

^13^C-NMR ((CD_3_)_2_CO, 125.76 MHz): δ [ppm] = 199.8 (C-11, >C=O), 178.8 (C-24, COOH), 175.1 (C-35, COOH (glutaroyl)), 173.7 (C-31, >C=O (glutaroyl)), 165.8 (C-13, H>C=C<), 132.2 (C-12, H>C=C<), 74.9 (C-3, HO>CH–), 62.0 (C-9, >CH–), 60.8 (C-18, >CH–), 52.0 (C-5, >CH–), 48.1 (C-4, >C<), 46.7 (C-8, >C<), 45.5 (C-14, >C<), 42.7 (C-22, –CH_2_–), 41.1 (C-19, >CH–), 40.9 (C-20, >CH–), 39.2 (C-10, >C<), 36.4 (C-1, –CH_2_–), 35.7 (C-17, >C<), 35.1 (C-34, –CH_2_– (glutaroyl)), 34.5 (C-7, –CH2–), 34.3 (C-32, –CH_2_– (glutaroyl)), 32.6 (C-21, -CH_2_-), 30.2 (C-28, -CH_3_), 29.2 (C-16, -CH_2_-), 28.9 (C-15, –CH2–), 25.3 (C-23, –CH3), 25.2 (C-2, –CH2–), 22.4 (C-30, –CH3), 22.1 (C-33, –CH_2_– (glutaroyl)), 21.9 (C-27, –CH_3_), 21.9 (C-6, –CH_2_–), 19.9 (C-26, –CH_3_), 18.7 (C-29, –CH_3_), 14.8 (C-25, –CH_3_).

MS (EI, 70 eV): *m*/*z* (%) = 584 (4) [M]^+^, 452 (15), 408 (18), 273 (19), 232 (39), 228 (60), 182 (100). HRMS (EI, 70 eV): calculated: 584.3713 for C_35_H_52_O_7_; found: 584.3713. [α]^D^: +67.7° (c = 0.64, acetone). m.p. 139–142 °C (dec).

3-*O*-Carboxymethyl-β-boswellic acid **26**. β-BA **3** (300 mg, 0.66 mmol) was dissolved in dry THF (2 mL). This solution was added dropwise to a suspension of NaH (154 mg, 6.4 mmol) in dry THF (3 mL). After 5 min a solution of 2-chloroacetic acid (242 mg, 2.56 mmol) in dry THF (1.6 mL) was added dropwise and the mixture was refluxed overnight. After cooling to rt, the reaction mixture was acidified with 1 N HCl to pH 3 and extracted three times with diethyl ether (ca. 10 mL each). The combined organic extracts were washed with H_2_O and brine and were dried with MgSO_4_. After filtering the MgSO_4_ the solvent is removed in vacuo. The white solid is further purified by flash chromatography with pentane/diethylether 1:1 + 1% HOAc and a second flash chromatography with dichloromethane/MeOH 15:1 yielding 157 mg of **26** as white solid.

^1^H-NMR (CDCl_3_, 500.13 MHz): δ [ppm] = 5.14 (t, *J =* 3.4 Hz, 1H, H-12), 4.21 (d, *J =* 16.9 Hz, 1H, H-31 (carboxymethyl)), 4.08 (d, *J =* 16.9 Hz, 1H, H-31 (Carboxymethyl)), 3.78 (bs, 1H, H-3), 2.06–1.96 (m, 2H, H-2β, 16α), 1.95–1.88 (m, 2H, H-11α, H-11β), 1.88–1.79 (m, 2H, H-6β, H-15β), 1.79–1.67 (m, 2H, H-2α, H-6α), 1.62–1.56 (m, 1H, H-9), 1.56–1.45 (m, 3H, H-1β, H-5, H-7α), 1.45–1.42 (m, 1H, H-22α), 1.42 (s, 3H, H-23), 1.42–1.36 (m, 2H, H-7β, H-21β), 1.35–1.30 (m, 2H, H-18, H-19), 1.30–1.20 (m, 3H, H-1α, H-21α, H-22β), 1.10 (s, 3H, H-27), 1.04 (s, 3H, H-26), 1.04–0.98 (m, 1H, H-15α), 0.92 (d, *J =* 6.5 Hz, 3H, H-30), 0.91 (s, 3H, H-25), 0.91–0.88 (m, 2H, H-16β, H-20), 0.80 (s, 3H, H-28), 0.79 (d, *J =* 5.7 Hz, 3H, H-30).

^13^C-NMR (CDCl_3_, 125.76 MHz): δ [ppm] = 183.3 (C-24, COOH), 174.6 (C-32, COOH (carboxymethyl)), 139.6 (C-13, H>C=C<), 124.4 (C-12, H>C=C<), 80.1 (C-3, HO>CH–), 66.2 (C-31, –CH_2_– (carboxymethyl)), 59.2 (C-18, >CH–), 49.9 (C-5, >CH–), 47.9 (C-4, >C<), 46.7 (C-9, >CH–), 42.3 (C-14, >C<), 41.5 (C-22, –CH_2_–), 40.0 (C-8, >C<), 39.7 (C-19, >CH–), 39.6 (C-20, >CH–), 37.5 (C-10, >C<), 34.1 (C-1, –CH_2_–), 33.8 (C-17, >C<), 33.0 (C-7, –CH_2_–), 31.3 (C-21, –CH_2_–), 28.8 (C-28, -CH_3_), 28.1 (C-16, –CH_2_–), 26.5 (C-15, –CH_2_–), 24.2 (C-23, –CH_3_), 23.4 (C-11, –CH_2_–), 23.3 (C-27, –CH_3_), 21.4 (C-2, –CH_2_–), 21.4 (C-30, –CH_3_), 19.6 (C-6, –CH_2_–), 17.5 (C-29, –CH_3_), 16.9 (C-26, –CH_3_), 13.6 (C-25, –CH_3_).

MS (CI, 150 eV): *m*/*z* (%) = 514 (76) [M]^+^, 499 (12), 470 (29), 439 (100), 393 (38), 379 (8), 218 (8). HRMS (CI, 150 eV): calculated: 514.3699 for C_32_H_50_O_5_; found: 514.3679. [α]^D^: +61.7° (c = 0.72, methanol). m.p. 265–269 °C (dec).

3-*O*-Carboxymethyl-11-keto-β-boswellic acid **27** was prepared analogously to **26**.

^1^H-NMR (CDCl_3_, 500.13 MHz): δ [ppm] = 5.55 (s, 1H, H-12), 4.20 (d, *J =* 16.8 Hz, 1H, H-31 (carboxymethyl)), 4.07 (d, *J =* 16.9 Hz, 1H, H-31 (carboxymethyl)), 3.78 (bs, 1H, H-3), 2.54–2.46 (m, 1H, H-1β), 2.41 (s, 1H, H-9), 2.12–2.04 (m, 2H, H-2β, H-16α), 1.94–1.82 (m, 2H, H-6β, H-15β), 1.77–1.62 (m, 3H, H-2α, H-6α, H-7α), 1.55–1.42 (m, 4H, H-5, H-7β, H-18, H-22α), 1.42 (s, 3H, H-23), 1.42-1.32 (m, 2H, H-19, H-21β), 1.32 (s, 3H, H-27), 1.32–1.18 (m, 4H, H-1α, H-15α, H-21α, H-22β), 1.18 (s, 3H, H-26), 1.14 (s, 3H, H-25), 1.05–0.98 (m, 1H, H-16β), 0.94 (bs, 4H, H-20, H-30), 0.82 (s, 3H, H-28), 0.80 (d, *J =* 6.3 Hz, 3H, H-29).

^13^C-NMR (CDCl_3_, 125.76 MHz): δ [ppm] = 199.5 (C-11, >C=O), 182.4 (C-24, COOH), 174.2 (C-32, COOH (carboxymethyl)), 165.3 (C-13, H>C=C<), 130.4 (C-12, H>C=C<), 79.9 (C-3, HO>CH–), 66.1 (C-31, –CH_2_– (carboxymethyl)), 60.3 (C-9, >CH–), 59.0 (C-18, >CH–), 49.6 (C-5, >CH–), 47.7 (C-4, >C<), 45.1 (C-8, >C<), 43.8 (C-14, >C<), 40.9 (C-22, –CH_2_–), 39.3 (C-19, >CH–), 39.3 (C-20, >CH–), 37.4 (C-10, >C<), 34.0 (C-1, –CH_2_–), 34.0 (C-17, >C<), 32.8 (C-7, –CH_2_–), 30.9 (C-21, –CH_2_–), 28.9 (C-28, –CH_3_), 27.5 (C-16, –CH_2_–), 27.2 (C-15, –CH_2_–), 24.4 (C-23, –CH_3_), 21.1 (C-2, –CH_2_–), 21.1 (C-30, –CH_3_), 20.6 (C-27, –CH_3_), 18.8 (C-6, –CH_2_–), 18.4 (C-26, –CH_3_), 17.4 (C-29, –CH_3_), 13.5 (C-25, –CH_3_).

MS (CI, 150 eV): *m*/*z* (%) = 529 (91) [M + H]^+^, 453 (13), 408 (7), 299 (5), 287 (7), 273 (100), 232 (97). HRMS (CI, 150 eV): calculated: 528.3467 for C_32_H_48_O_6_; found: 528.3459. [α]^D^: +94.4° (c = 0.91, chloroform). m.p. 215–217 °C (dec).

### 4.2. Cells and Cell Isolation

Neutrophils were isolated from peripheral blood (University Hospital Tübingen, Germany), collected from fasted healthy adult donors that were informed about the aim of the study and gave written consent. Leukocyte concentrates were obtained by centrifugation (4000× *g*, 20 min, 20 °C) of heparinized blood preparation, and neutrophils were immediately isolated as described before [30]. Briefly, leukocyte concentrates were subjected to dextran sedimentation and centrifuged on lymphocyte separation medium (LSM 1077, PAA, Coelbe, Germany). For isolation of pelleted neutrophils, remaining erythrocytes were removed by hypotonic lysis, washed twice with ice-cold phosphate-buffered saline (PBS) and finally resuspended in PBS buffer containing 0.1% glucose and CaCl_2_ (1 mM) (PGC buffer).

### 4.3. Expression and Purification of Human Recombinant 5-LO

Human recombinant 5-LO was expressed in *Eschericha coli* BL21 transformed with pT3-5-LO plasmid at 30 °C overnight, as described elsewhere [31]. Cells were lysed in lysis buffer containing triethanolamine (50 mM, pH 8.0), EDTA (5 mM), phenylmethanesulfonyl fluoride (1 mM), soybean trypsin inhibitor (60 µg/mL), dithiothreitol (2 mM), and lysozyme (1 mg/mL), and homogenized by sonification (3 × 15 s). 5-LO was then purified from 40,000× *g* supernatant (20 min, 4 °C) using an ATP-agarose column (Sigma-Aldrich, Deisenhofen, Germany), diluted with PBS buffer containing 1 mM EDTA, and immediately used for 5-LO activity assays. 

### 4.4. Determination of 5-LO Activity in a Cell-Free Assay

To determine 5-LO activity, aliquots of purified 5-LO (0.5 µg 5-LO in 1 mL PBS plus 1 mM EDTA) were pre-incubated with the test compounds or vehicle (0.1% dimethyl sulfoxide, DMSO) on ice for 15 min, pre-warmed for 30 s at 37 °C in a water bath, and then stimulated with 20 µM AA and CaCl_2_ 2 mM for 10 min at 37 °C. The reaction was stopped with one volume of ice-cold methanol and 5-LO products (including all-*trans*-isomers of LTB_4_ and 5-hydroperoxyeicosatetraenoic acid (5-HpETE), as well as its corresponding alcohol 5-hydroxyeicosatetraenoic acid (5-HETE)) were analyzed by reversed phase (RP)-HPLC (Elite LaChrom, VWR, Radnor, PA, USA) as previously described [32]. In brief, acidified PBS and 200 ng of internal PGB_1_ standard were added and solid phase extraction using C18 RP-columns (100 mg, UCT, Bristol, PA, USA) was performed. After elution with methanol, samples were analyzed by RP-HPLC using a C-18 Radial-PAK column (Waters, Eschborn, Germany). 

### 4.5. Determination of 5-LO Product Formation in Neutrophils

5-LO product formation in intact human neutrophils was performed using 5 × 10^6^ freshly isolated cells that were resuspended in 1 mL PGC buffer. Cells were pre-incubated with the test compounds or vehicle (0.1% DMSO) at 37 °C for 15 min prior to stimulation with 2.5 µM Ca^2+^-ionophore A23187 for 10 min (37 °C). 5-LO product formation was stopped by addition of one volume of ice-cold methanol, samples were subjected to solid phase extraction after addition of 200 ng PGB_1_ as internal standard, and 5-LO products (LTB_4_, its *trans*-isomers and 5-HETE) were analyzed by RP-HPLC as described above. 

### 4.6. Determination of Cathepsin G Activity in a Cell-Free Assay

Commercially available purified cathepsin G (0.2 µg) from human neutrophils (Calbiochem, La Jolla, CA, USA) was mixed with test compounds or DMSO (vehicle control) in 200 µL 0.1 M HEPES pH 7.4, 0.5 M NaCl, and 10% DMSO in a 96-well plate (Greiner Bio-One, Frickenhausen, Germany) and pre-incubated for 20 min at 25 °C. Then, *N*-Suc–Ala–Ala–Pro–Phe–pNA (Suc–AAPF–pNA) (1 mM) as substrate for cathespin G was added, and the absorbance was measured at 410 nm at 25 °C using a Victor^2^ plate reader (PerkinElmer, Waltham, MA, USA). The enzymatic activity was determined by the progress curve method, and cathepsin G activity is given as percentage of the vehicle (DMSO) control. 

### 4.7. Statistics

Results are presented as mean ± standard error of the mean (SEM) out of *n* independent experiments, where *n* represents the number of experiments performed on different days or with different donors. IC_50_ values were calculated from at least 5 different concentrations using a nonlinear regression interpolation of semi-logarithmic graphs in GraphPad Prism (Graphpad Software Inc., San Diego, CA, USA). Statistical evaluation was performed by two-tailed student *t*-test for single comparisons. *P*-values < 0.05 were considered as significant.

## Figures and Tables

**Table 1:** Effects of triterpene acids on 5-lipoxygenase (5-LO) and cathepsin G.

Cmpd.	Structure			5-LO Activity [% Control] (IC_50_ in µM)		Cat. G Activity [% Control] (IC_50_ in µM)
				cell-based	cell-free	
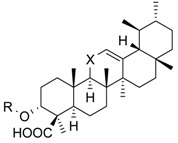						
**1**	R = –CO–CH_3_ X = C=O			23.1 ± 9.8 ** (3.0 µM)	40.7 ± 10.8 * (3.0 µM)	14.8 ± 0.9 *** (0.6 µM)
**2**	R = –H X = C=O			25.7 ± 7.3 ** (3.5 µM)	31.1 ± 5.0 *** (4.6 µM)	29.4 ± 3.5 *** (4.1 µM)
**3**	R = –H X = CH_2_			53.0 ± 7.6 *	78.7 ± 12.6	27.7 ± 1.8 *** (0.5 µM)
**4**	R = –CO–CH_3_ X = CH_2_			n.i.	73.5 ± 13.3	28.1 ± 4.9 *** (2.0 µM)
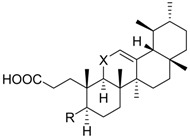						
**5**	R =	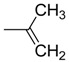		74.5 ± 3.7 **	n.i.	69.8 ± 8.0
	X = CH_2_					
**6**	R =	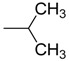		n.i.	n.i.	70.4 ± 3.9 **
	X = CH_2_					
**7**	R =	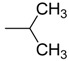		7.1 ± 3.3 *** (4.3 µM)	73.0 ± 8.1	53.8 ± 8.1 *
	X = C=O					
**8**	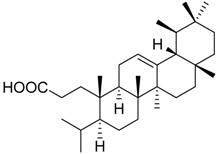			n.i.	89.1 ± 7.5	59.2 ± 4.0 **
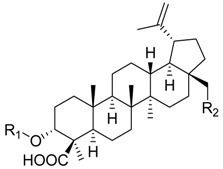						
**9**	R_1_ = –H R_2_ = –H			36.2 ± 5.1 *** (4.0 µM)	76.6 ± 12.2	58.1 ± 3.6 **
**10**	R_1_ = –CO–CH_3_ R_2_ = –H			88.7 ± 6.7	n.i.	35.9 ± 2.5 *** (7.5 µM)
**11**	R_1_ = –H R_2_ = –OH			24.0 ± 7.2 ** (4.6 µM)	52.7 ± 7.1 **	68.1 ± 6.5 *
**12**	R_1_ = –CO–CH_3_ R_2_ = –OH			28.4 ± 11.1 * (5.1 µM)	41.8 ± 3.4 *** (8.3 µM)	87.2 ± 2.5 *
**13**	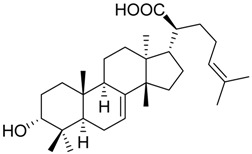			8.2 ± 2.2 *** (2.9 µM)	89.6 ± 8.5	72.3 ± 3.6 **
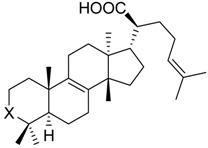						
**14**	X =	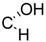	(*S*)	3.1 ± 0.6 *** (1.1 µM)	66.0 ± 15.1	73.0 ± 7.3
**15**	X =	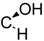	(*R*)	5.2 ± 1.0 *** (3.0 µM)	64.1 ± 12.4	80.1 ± 6.2
**16**	X = C=O			37.8 ± 2.4 *** (7.1 µM)	77.1 ± 8.7	53.1 ± 5.5 **
**17**	X =	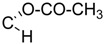		75.5 ± 2.1 **	75.6 ± 3.0**	66.7 ± 4.5 **
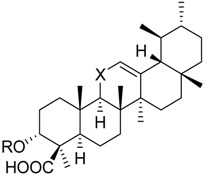						
**18**	X =	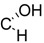	(*R*)	18.3± 2.7 *** (2.8 µM)	72.8 ± 7.1	62.9 ± 6.6 *
	R = –H					
**19**	X =	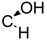	(S)	58.2 ± 0.9 ***	87.2 ± 1.8 **	82.7 ± 9.0
	R = –H					
**20**	X = CH_2_ R = –CO–COOH			77.3 ± 5.7 *	n.i.	38.1 ± 6.4 *** (4.7 µM)
**21**	X = C=O R = –CO–COOH			70.3 ± 6.9 *	n.i.	n.i.
**22**	X = CH_2_ R = –CO– (CH_2_)_2_–COOH			60.3 ± 8.0 *	85.9 ± 11.1	59.4 ± 7.6 *
**23**	X = C=O R = –CO– (CH_2_)_2_–COOH			80.8 ± 6.8	75.0 ± 4.8*	n.i.
**24**	X = CH_2_ R = –CO– (CH_2_)_3_–COOH			65.3 ± 8.0 *	n.i.	24.5 ± 1.7 *** (3.6 µM)
**25**	X = C=O R = –CO– (CH_2_)_3_–COOH			n.i.	n.i.	49.1 ± 11.0 * (8.6 µM)
**26**	X = CH_2_ R = –CH_2_–COOH			57.6 ± 5.3 **	78.1 ± 7.4	28.8 ± 7.4 ** (3.4 µM)
**27**	X = C=O R = –CH_2_–COOH			68.4 ± 2.1 ***	n.i.	n.i.

Residual activity (% control) and IC_50_ values (µM) are given as mean ± SEM of single determinations obtained in 3–4 independent experiments. (*) *P* < 0.05, (**) *P* < 0.01, (***) *P* < 0.001; student *t*-test. n.i., no inhibition.
